# Comparison of M.I.C.E. and Etest with CLSI Agar Dilution for Antimicrobial Susceptibility Testing against Oxacillin-Resistant *Staphylococcus* spp

**DOI:** 10.1371/journal.pone.0094627

**Published:** 2014-04-14

**Authors:** Eloiza H. Campana, Cecilia G. Carvalhaes, Bruna Nonato, Antonia M. de. O. Machado, Ana C. Gales

**Affiliations:** 1 Laboratório Alerta, Disciplina de Infectologia, Universidade Federal de São Paulo, São Paulo - SP, Brazil; 2 Laboratório Central do Hospital São Paulo, Universidade Federal de São Paulo, São Paulo - SP, Brazil; The University of Hong Kong, Hong Kong

## Abstract

**Objective:**

The main objective of this study was to comparatively evaluate the performance of M.I.C.E. and Etest methodologies to that of agar dilution for determining the antimicrobial susceptibility profile of oxacillin-resistant *Staphylococcus* spp.

**Methods:**

A total of 100 oxacillin-resistant *Staphylococcus* spp. isolates were collected from hospitalized patients at a teaching hospital. Antimicrobial susceptibility testing for vancomycin, teicoplanin and linezolid was performed using the reference CLSI agar dilution method (2009), Etest and M.I.C.E. methodologies. The MIC values were interpreted according to CLSI susceptibility breakpoints and compared by regression analysis.

**Results:**

In general, the essential agreement (±1-log_2_) between M.I.C.E. and CLSI agar dilution was 93.0%, 84.0% and 77.0% for linezolid, teicoplanin and vancomycin, respectively. Essential agreement rates between M.I.C.E. and Etest were excellent (>90.0%) for all antibiotics tested. Both strips (M.I.C.E. and Etest) yielded two very major errors for linezolid. Unacceptable minor rates were observed for teicoplanin against CoNS and for vancomycin against *S. aureus*.

**Conclusions:**

According to our results, linezolid and teicoplanin MICs against all staphylococci and *S. aureus*, respectively, were more accurately predicted by M.I.C.E. strips. However, the Etest showed better performance than M.I.C.E. for predicting vancomycin MICs against all staphylococci. Thus, microbiologists must be aware of the different performance of commercially available gradient strips against staphylococci.

## Introduction

Determination of vancomycin minimum inhibitory concentration (MIC) has been of crucial importance to guide antimicrobial therapy against staphylococcal infections since intermediate resistance to vancomycin has not been accurately detected by disc diffusion [1]. In addition, treatment failures have been reported when vancomycin is prescribed for treatment of oxacillin-resistant *Staphylococcus aureus* (ORSA) infections, especially for strains exhibiting vancomycin MICs, ≥ 2 µg/mL. Studies have suggested that vancomycin treatment success rates are indirectly proportional to vancomycin MICs, i.e., vancomycin treatment success decreases as the MIC of the ORSA strains increases [2]. In this manner, alternative therapeutic drugs such as linezolid have been considered for treatment of ORSA infections. Linezolid has activity against clinically significant gram-positive cocci, including ORSA and coagulase-negative *Staphylococcus* (CoNS). The Clinical Laboratory Standards Institute (CLSI) document M100-S22 [3] recommends the determination of the linezolid MIC for isolates categorized as non-susceptible by disc-diffusion methodology. Therefore, the Oxoid M.I.C.Evaluator™ (M.I.C.E.) (Thermo Fisher Scientific, Basingstoke, UK) methodology represents a rapid alternative for determining vancomycin and linezolid MICs.

In a previous study, we had compared the vancomycin MICs determined by M.I.C.E. with those obtained by CLSI broth microdilution (BMD) and observed that the vancomycin MICs values determined by M.I.C.E. were higher than those obtained by BMD [4]. A similar finding was latter reported by Rennie *et al*. [5]. We thought these results could have resulted from the different techniques, gradient agar diffusion vs. BMD, employed. The clinical relevance of determining vancomycin MIC by a reliable technique motivated us to perform the current study, where the performance of M.I.C.E. was comparatively evaluated with those of CLSI agar dilution and Etest (AB bioMérieux, Marcy l'Étoile, France).

## Materials and Methods

### Ethical Statement

Ethical approval was not required because the study was conducted as part of surveillance control management. Written informed consent was not required because patients received routine clinical care, and there were no additional specimens collected or study-specific interventions. Patient records/information was anonymized and de-identified prior to analysis.

### Bacterial Strains

A total of 100 clinical oxacillin-resistant *Staphylococcus* spp. isolates (50 *S. aureus* and 50 coagulase-negative staphylococci; CoNS) were collected from hospitalized patients at a Brazilian teaching hospital located in the city of São Paulo. All bacterial isolates were recovered from blood culture. Only a single isolate per patient was evaluated. *S. haemolyticus* (58%) was the most frequent specie among CoNS, followed by *S. hominis* (30%) and *S. epidermidis* (12%). No *Staphylococcus lugdunensis* isolate was identified in this collection. Confirmation of species identification was performed by MALDI-TOF mass spectrometry (Bruker Daltonics, Bremen, Germany) after confirming that the cultures were pure and possessed identical colony morphologies.

### Antimicrobial Susceptibility Testing (AST)

AST was performed against vancomycin, teicoplanin and linezolid by M.I.C.E., Etest (gradient methods), and agar dilution (reference method) according to CLSI guidelines or respective manufacturers [3], [6]. Antimicrobial powders were purchased from Sigma-Aldrich (St. Louis, MO, USA). The Etest and M.I.C.E. MICs were determined as the value at which the elliptical growth margin intersected the strips, except for linezolid. For this antimicrobial agent, different manufacturer's recommendations have been established for MIC reading, i.e. 90% vs. 80% of the growth inhibition by Etest and M.I.C.E., respectively. MICs were rounded up to the next higher twofold dilution for comparison purposes. Quality control of susceptibility testing was performed by testing *S. aureus* ATCC 29213 and *Enterococcus faecium* ATCC 29212 with results within the CLSI expected ranges for all antimicrobials and AST methods tested. The MICs were read by three independent observers with no discordant MICs readings.

### Statistical Analysis

The results of the MICs obtained by agar dilution, Etest and M.I.C.E. techniques were analyzed and compared by regression analysis using the Statistical Package for the Social Sciences, version 17.0 (SPSS Inc., Chicago, IL).

Essential agreement was defined when the result of the MICs obtained by Etest or M.I.C.E. ranged ±1-log_2_ dilution of those obtained by agar dilution (reference method). Differences of ≥2-log_2_ dilutions were considered as discordant results. Categorical agreement was defined as test results within the same susceptibility category. Errors were ranked as follows: very major errors, false susceptible results by gradient methods; major errors, false resistant results produced by gradient methods; and minor errors, intermediate by reference method and susceptible or resistant by the gradient methods or intermediate by the gradient methods and susceptible or resistant by the reference method. Acceptable error levels were ≤1.5% for very major errors, ≤3% for major errors and 10% for minor errors as recommended [7].

## Results

In comparison to agar dilution, M.I.C.E. methodology yielded essential and categorical agreement rates of 77.0%/83.0%, 84.0%/87.0%, and 93.0%/98.0% for vancomycin, teicoplanin, and linezolid, respectively. When these rates were analyzed according to the staphylococcal species, lower essential and categorical agreement vancomycin rates were observed for *S. aureus* than CoNS isolates (66.0%/68.0% and 88.0%/98.0%, respectively), as shown in Table 1. In contrast, for teicoplanin, lower essential and categorical agreement rates were observed for CoNS (80.0%/74.0%) than *S. aureus* (88.0%/100%). When testing linezolid, a variation in the essential and categorical agreement rates was also observed according to staphylococcal species (98.0%/96.0% and 88.0%/100% for *S. aureus* and CoNS, respectively). Essential and categorical agreement rates for teicoplanin against CoNS varied according to CoNS species. These rates were lower for *S. haemolythicus* than those observed for *S. epidermidis* and *S. hominis*. Most discordant results led to occurrence of minor errors (16.0% and 12.0% for vancomycin and teicoplanin against *S. aureus* and CoNS, respectively). One (2.0%) major error and two (4.0%) very major errors were observed for vancomycin and linezolid against *S. aureus*, respectively.

**Table 1:** Essential and categorical agreement rates between gradient diffusion tests (M.I.C.E. and Etest) against *Staphylococcus* spp.

Diffusion Test	M.I.C.E.		Etest	
	Essential	Categorical	Essential	Categorical
**Vancomycin**				
***S. aureus***	**66.0%**	**68.0%**	**92.0%**	**98.0%**
*S. epidermidis*	83%	100,0%	100%	83%
*S. haemolythicus*	90%	97,0%	97%	97%
*S. hominis*	87%	100,0%	100%	93%
**CoNS**	**88.0%**	**98.0%**	**98.0%**	**94.0%**
**General^a^**	**77.0%**	**83.0%**	**95.0%**	**96.0%**
**Teicoplanin**				
***S. aureus***	**88.0%**	**100%**	**82.0%**	**100%**
*S. epidermidis*	100%	100%	100%	83%
*S. haemolythicus*	76%	66%	72%	72%
*S. hominis*	80%	80%	67%	80%
**CoNS**	**80.0%**	**74.0%**	**74.0%**	**76.0%**
**General^a^**	**84.0%**	**87.0%**	**78.0%**	**88.0%**
Linezolid				
***S. aureus***	**98.0%**	**96.0%**	**66.0%**	**96.0%**
*S. epidermidis*	100%	100%	83%	100%
*S. haemolythicus*	90%	100%	72%	100%
*S. hominis*	80%	100%	47%	100%
**CoNS**	**88.0%**	**100%**	**66.0%**	**100%**
**General^a^**	**93.0%**	**98.0%**	**66.0%**	**98.0%**

a. *S. aureus* and CoNS species.

The essential and categorical agreement rates obtained by Etest compared to agar dilution were 95.0%/96.0%, 78.0%/88.0%, and 66.0%/98.0% for vancomycin, teicoplanin, and linezolid, respectively. A trend for lower linezolid MICs was observed with Etest against both species analyzed. According to staphylococcal species no significant variation in the essential and categorical agreement rates were observed for vancomycin (92.0%/98.0% and 98.0%/94.0% for *S. aureus* and CoNS, respectively). However, lower essential and categorical agreement rates were observed for CoNS (74.0%/76.0%) than *S. aureus* (82.0%/100%) for teicoplanin. The majority of discordant results led to occurrence of minor errors (11.0% and 6.0% for teicoplanin and vancomycin, respectively). One (2.0%) major error against CoNS and two (4.0%) very major errors against *S. aureus* were observed for teicoplanin and linezolid, respectively.

An excellent concordance was observed between M.I.C.E. and Etest MIC results, which yielded essential agreement rates of 94.0%, 100.0% and 93.0% for vancomycin, teicoplanin, and linezolid, respectively. However, an unacceptable minor error rate (18.0%) was detected for vancomycin due to a trend of even higher MIC values by M.I.C.E. strips.

## Discussion

We observed a trend for higher vancomycin MICs for both strips, Etest and M.I.C.E. This finding is corroborated by previous studies performed under CLSI recommendations independent of methodology tested, BMD or agar dilution [4], [5]. In general, vancomycin MICs determined by M.I.C.E. showed a 1-log_2_ dilution higher than those of Etest [4], [5]. It is important to notice that vancomycin CLSI susceptibility breakpoints for *S. aureus* (≤2 µg/mL) are 1-log_2_ dilution lower than those of CoNS (≤4 µg/mL). It could be one of the reasons for the better performance of M.I.C.E. strips against CoNS.

To the best of our knowledge, only five studies evaluating M.I.C.E. methodology have been published to date [4], [5], [8]–[10]. Only three of them evaluated *Staphylococcus* spp., as shown in Table 2. Our results are in agreement with those studies already published, except for those published by Mushtaq *et al*. [8]. These distinct findings could be due to the medium employed for AST, since Mushtaq *et al*. [8] tested Iso-Sensitest agar according to the British Society for Antimicrobial Chemotherapy (BSAC) guidelines. The distinct set of *Staphylococcus* isolates tested could also have contributed to the different results.

**Table 2:** Essential agreement rates between gradient diffusion tests (M.I.C.E. and Etest) and reference techniques (agar and broth microdilution) against *Staphylococcus* spp.

Method	Agar dilution				Broth microdilution			
Guideline	CLSI^a^		BSAC^b^		CLSI^a^		CLSI^a^	
Antimicrobial	This study		Mushtaq *et al.* 2010		Carvalhaes *et al.* 2011		Rennie *et al.* 2012	
	M.I.C.E.	Etest	M.I.C.E.	Etest	M.I.C.E.	Etest	M.I.C.E.	Etest
**Vancomycin**	77.0%	95.0%	95.6%	96.2%	72.5%	90.0%	61.0%	84.0%
**Teicoplanin**	84.0%	78.0%	-	-	-	-	-	-
**Linezolid**	93.0%	66.0%	99.3%	99.3%	97.5%	100%	74.0%	52.0%
**N of isolates**	100		154		40		157	

a. CLSI, Clinical Laboratory Standards Institute;

b. BSAC, British Society for Antimicrobial Chemotherapy.

The present study was the only one to evaluate the performance of M.I.C.E. for testing teicoplanin against *Staphylococcus* spp. Despite of not observing a trend for higher teicoplanin MIC values, the categorical agreement for both strips (M.I.C.E. and Etest) compared to agar dilution against CoNS was lower than 80%. When the M.I.C.E. results for teicoplanin against CoNS were evaluated, the categorical agreement rate (74.0%) remained lower than that of essential agreement (80.0%). The breakpoints for interpreting the category of susceptibility to teicoplanin against CoNS vary between CLSI (susceptible ≤ 8 µg/mL; resistant ≥ 32 µg/mL) and EUCAST (susceptible ≤ 4 µg/mL; resistant > 4 µg/mL). This fact could justify the high rates of minor (24.0%) and major errors (14.0%) obtained by interpreting this compound by these respective guidelines, and reemphasize the need for revision of the current teicoplanin breakpoints against CoNS.

Different results have been reported for linezolid by different authors. It might be due to the use of distinct reading criteria (80% vs 90% of growth inhibition for M.I.C.E. and Etest, respectively). In the present study, a trend for lower linezolid MICs was noticed for Etest, in agreement with a previous report [5]. In general, linezolid MICs determined by Etest showed a 1-log_2_ dilution higher than those of agar dilution; however, this trend did not result in changes in the categorization of the isolates.

If M.I.C.E. results were interpreted according to the European Committee on Antimicrobial Susceptibility Testing (EUCAST) [11] breakpoints, no variation in the categorical agreement rates would be observed for vancomycin and linezolid against *S. aureus* and CoNS, as shown in Table 3. However, the categorical agreement rate for teicoplanin against *S. aureus* would drop from 100% to 68.0%, while it would increase from 74.0% to 80.0% against CoNS. In addition, by applying the EUCAST breakpoints, the error rates would be unacceptable for vancomycin and teicoplanin against *S. aureus* and for teicoplanin against CoNS. These different rates could be consequent to the absence of an intermediate category by EUCAST.

**Table 3:** Essential and categorical agreement rates by applying CLSI and EUCAST breakpoints against *S. aureus* and CoNS.

Specie		*S. aureus*										CoNS									
Guideline		CLSI^a^					EUCAST^b^					CLSI^a^					EUCAST^b^				
Antimicrobial		Categorical Agreement	Minor Errors		Major Errors	Very Major Errors	Categorical Agreement	Minor Errors		Major Errors	Very Major Errors	Categorical Agreement	Minor Errors		Major Errors	Very Major Errors	Categorical Agreement	Minor Errors		Major Errors	Very Major Errors
**M.I.C.E.**	**Vancomycin**	68.0%	30.0%		2.0%	0%	68.0%	-		32.0%	0%	98.0%	2.0%		0%	0%	98.0%	-		2.0%	0%
	**Teicoplanin**	100%	0%		0%	0,0%	68.0%	-		28.0%	4.0%	74.0%	24.0%		0%	2%	80.0%	-		14.0%	6.0%
	**Linezolid**	96.0%	0%		0%	4.0%	96.0%	-		0%	4.0%	100%	0%		0%	0%	100%	-		0%	0%
**Etest**	**Vancomycin**	98.0%	2.0%		0%	0%	98.0%	-		2.0%	0%	94.0%	6.0%		0%	0%	98.0%	-		2.0%	0%
	**Teicoplanin**	100%	0%		0%	0%	62.0%	-		34.0%	4.0%	76.0%	22.0%		2%	0%	72.0%	-		22.0%	6.0%
	**Linezolid**	96.0%	0%		0%	4.0%	96.0%	-		0%	4.0%	100%	0%		0%	0%	100%	-		0%	0%
**Susceptibility profile**		**S%**		**I%**	**R%**		**S%**		**I%**	**R%**		**S%**		**I%**	**R%**		**S%**		**I%**	**R%**	
**Vancomycin**		100%		0%	0%		100%		-	0%		100%		0%	0%		100%		-	0%	
**Teicoplanin**		100%		0%	0%		96%		-	4%		72%		12%	16%		58%		-	42%	
**Linezolid**		96%		0%	4%		96%		-	4%		100%		0%	0%		100%		-	0%	

CLSI, Clinical Laboratory Standards Institute. Breakpoints: Vancomicyn: *S. aureus* S≤2, I 4-8, R≥16, CoNS S≤4, I 8-16, R≥32; Teicoplanin: S≤8, I 16, R≥32; Linezolid: S≤4, R≥8.

EUCAST, European Committee on Antimicrobial Susceptibility Testing. Breakpoints: Vancomicyn: *S. aureus* S≤2, R>2, CoNS S≤4, R>4; Teicoplanin: *S. aureus* S≤2, R>2, CoNS S≤4, R>4; Linezolid: S≤4, R>4.

According to our results, linezolid and teicoplanin MICs against all staphylococci and *S. aureus*, respectively, were more accurately predicted by M.I.C.E. strips. However, the Etest showed better performance than M.I.C.E. for predicting vancomycin MICs against all staphylococci. Thus, microbiologists must be aware of the different performance of commercially available gradient strips against staphylococci.
